# Prevalence of Stroke — Behavioral Risk Factor Surveillance System, United States, 2011–2022

**DOI:** 10.15585/mmwr.mm7320a1

**Published:** 2024-05-23

**Authors:** Omoye E. Imoisili, Alina Chung, Xin Tong, Donald K. Hayes, Fleetwood Loustalot

**Affiliations:** 1 Division for Heart Disease and Stroke Prevention, National Center for Chronic Disease Prevention and Health Promotion, CDC.

**Summary**

**What is already known about this topic?**

Stroke is the fifth leading cause of death in the United States and a leading cause of long-term disability. During 2006–2010, stroke prevalence decreased by 3.7%.

**What is added by this report?**

From 2011–2013 to 2020–2022, U.S. stroke prevalence increased by 7.8%. Increases occurred among adults aged 18–64 years; females and males; non-Hispanic Black or African American (Black), non-Hispanic White, and Hispanic or Latino (Hispanic) adults; and adults with less than a college degree. Stroke prevalence was higher among adults aged ≥65 years; non-Hispanic American Indian or Alaska Native, non-Hispanic Native Hawaiian or Pacific Islander, and Black adults; and adults with lower education. Stroke prevalence decreased in the District of Columbia and increased in 10 states.

**What are the implications for public health practice?**

Initiatives to promote knowledge of the signs and symptoms of stroke, and identification of disparities in stroke prevalence, might help effectively focus interventions to improve stroke prevention and treatment.

## Abstract

Stroke was the fifth leading cause of death in the United States in 2021, and cost U.S. residents approximately $56.2 billion during 2019–2020. During 2006–2010, self-reported stroke prevalence among noninstitutionalized adults had a relative decrease of 3.7%. Data from the Behavioral Risk Factor Surveillance System were used to analyze age-standardized stroke prevalence during 2011–2022 among adults aged ≥18 years. From 2011–2013 to 2020–2022, overall self-reported stroke prevalence increased by 7.8% nationwide. Increases occurred among adults aged 18–64 years; females and males; non-Hispanic Black or African American (Black), non-Hispanic White (White), and Hispanic or Latino (Hispanic) persons; and adults with less than a college degree. Stroke prevalence was higher among adults aged ≥65 years than among younger adults; among non-Hispanic American Indian or Alaska Native, non-Hispanic Native Hawaiian or Pacific Islander, and Black adults than among White adults; and among adults with less than a high school education than among those with higher levels of education. Stroke prevalence decreased in the District of Columbia and increased in 10 states. Initiatives to promote knowledge of the signs and symptoms of stroke, and the identification of disparities in stroke prevalence, might help to focus clinical and programmatic interventions, such as the Million Hearts 2027 initiative or the Paul Coverdell National Acute Stroke Program, to improve prevention and treatment of stroke.

## Introduction

Stroke is a leading cause of morbidity in the United States and was the fifth leading cause of death in 2021.* The estimated direct and indirect cost of stroke in the United States was $56.2 billion during 2019–2020 (*1*). A report on stroke prevalence using Behavioral Risk Factor Surveillance System (BRFSS) data indicated that overall self-reported stroke prevalence among noninstitutionalized adults aged ≥18 years in all 50 states and the District of Columbia (DC) had a relative decrease of 3.7% during 2006–2010 (*2*). The current report used BRFSS data to assess stroke prevalence trends during 2011–2022 by sociodemographic characteristics and place of residence.

## Methods

### Data Source and Study Participants

BRFSS is a state-based surveillance system of noninstitutionalized U.S. civilian adults aged ≥18 years, administered in U.S. states and territories in coordination with CDC. Each year, health departments conduct a cross-sectional, random-digit–dialed landline and cellular telephone survey assessing health‐related risk behaviors and preventive health practices among approximately 400,000 residents in all 50 states, DC, Guam, Puerto Rico, and U.S. Virgin Islands. The analysis includes 5,225,987 respondents from the 50 states and DC during 2011–2022. Respondents with missing demographic data were excluded, as were those who responded, “Don’t know/Not sure” or “Refused” or who missed a response to the survey question, “Has a doctor or other health professional ever told you that you had a stroke?” Sample size ranged from 1,419,351 during 2011–2013 to 1,220,972 during 2020–2022. Median state and DC response rates ranged from 44.0% to 49.9%.^†^

### Definitions and Statistical Analysis

All data were self-reported. Participants who responded “yes” to “Has a doctor or other health professional ever told you that you had a stroke?” were defined as having had a stroke. Sociodemographic data included the following categories: age group (18–44, 45–64, and ≥65 years), sex (female and male), race and ethnicity (non-Hispanic American Indian or Alaska Native [AI/AN], non-Hispanic Asian [Asian], non-Hispanic Black or African American [Black], non-Hispanic Native Hawaiian or Pacific Islander [NH/PI], non-Hispanic White [White], and Hispanic or Latino [Hispanic] adults), education level (less than high school graduate, high school graduate or general educational development certificate, some college, and college graduate), and jurisdiction of residence. Prevalence estimates were age-standardized to the 2000 U.S. Census Bureau standard population, and analyses accounted for BRFSS complex sampling design. To obtain statistically stable estimates, annual data were combined to create four consecutive 3-year periods (2011–2013, 2014–2016, 2017–2019, and 2020–2022). Wald chi-square tests were used to assess statistical significance of the adjusted associations between each sociodemographic characteristic and stroke prevalence during 2020–2022. P-values were obtained through survey weighted logistic regression that included age group, sex, race and ethnicity, and education level. Both absolute (percentage point) and relative (percent) changes from 2011–2013 to 2020–2022, with 95% CIs, were calculated for age-standardized stroke prevalence by sociodemographic characteristics and by jurisdiction. R statistical software (version 4.1.2; R Foundation) was used to calculate 95% CIs by sampling normal distributions 5,000 times based on the age-standardized stroke prevalence and their SEs, defining the 95% CI as the 2.5 and 97.5 percentiles of calculations on the basis of those samples. SAS-callable SUDAAN (version 9.4; RTI International) was used to account for complex sampling design. A two-sided p-value <0.05 was considered statistically significant. This activity was reviewed by CDC, deemed not research, and was conducted consistent with applicable federal law and CDC policy.^§^

## Results

The age-standardized prevalence of self-reported stroke increased from 2.7% during 2011–2013, to 2.9% during 2020–2022, a 7.8% increase (Table 1). During 2020–2022, stroke prevalence was highest among adults aged ≥65 years (7.7%) and lowest among those aged 18–44 years (0.9%). By race and ethnicity, stroke prevalence was highest among AI/AN (5.3%), NH/PI (4.4%), and Black (4.3%) adults, and lowest among Asian adults (1.6%). Stroke prevalence among adults with less than a high school diploma was approximately three times that of adults who had graduated college. 

**TABLE 1:** Age-standardized prevalence* of stroke among noninstitutionalized adults aged ≥18 years,^†^ by selected characteristics — Behavioral Risk Factor Surveillance System, United States, 2011–2022^§^

Characteristic	% (95% CI)				p-value of differences across sociodemographic categories for 2020–2022^¶^	Change from 2011–2013 to 2020–2022	
	2011–2013	2014–2016	2017–2019	2020–2022		Percentage point (95% CI)	% (95% CI)
**Total**	**2.7 (2.7 to 2.8)**	**2.8 (2.8 to 2.9)**	**3.0 (2.9 to 3.0)**	**2.9 (2.9 to 3.0)**	**—**	**0.2 (0.1 to 0.3)**	**7.8 (4.9 to 10.8)**
**Age group, yrs**							
18–44	0.8 (0.7 to 0.8)	0.8 (0.8 to 0.9)	0.9 (0.8 to 0.9)	0.9 (0.8 to 1.0)	<0.01	0.1 (0 to 0.2)	14.6 (3.7 to 25.9)
45–64	3.3 (3.2 to 3.4)	3.6 (3.5 to 3.7)	3.9 (3.8 to 4.0)	3.8 (3.6 to 3.9)		0.5 (0.4 to 0.7)	15.7 (10.6 to 20.8)
≥65	7.7 (7.5 to 7.9)	7.7 (7.5 to 7.8)	7.9 (7.7 to 8.0)	7.7 (7.5 to 7.9)		0 (−0.3 to 0.3)	0 (−3.3 to 3.3)
**Sex**							
Female	2.7 (2.6 to 2.7)	2.8 (2.7 to 2.9)	2.9 (2.8 to 3.0)	2.9 (2.8 to 3.0)	0.65	0.2 (0.1 to 0.4)	9.3 (5.0 to 13.4)
Male	2.8 (2.7 to 2.8)	2.9 (2.8 to 2.9)	3.1 (3.0 to 3.2)	2.9 (2.8 to 3.0)		0.2 (0.1 to 0.3)	6.2 (2.1 to 10.5)
Race and ethnicity**							
AI/AN	5.4 (4.8 to 6.0)	5.7 (5.1 to 6.2)	6.2 (5.6 to 6.8)	5.3 (4.7 to 5.9)	<0.01	−0.1 (−1.0 to 0.7)	−2.0 (−16.4 to 15.3)
Asian	1.8 (1.4 to 2.2)	1.6 (1.2 to 1.9)	1.7 (1.4 to 2.1)	1.6 (1.2 to 2.0)		−0.2 (−0.7 to 0.3)	−11.8 (−35.9 to 18.5)
Black or African American	4.0 (3.8 to 4.2)	4.3 (4.1 to 4.4)	4.6 (4.3 to 4.8)	4.3 (4.1 to 4.5)		0.3 (0 to 0.6)	7.8 (0.4 to 15.5)
NH/PI	2.9 (1.6 to 4.2)	3.6 (2.4 to 4.7)	3.9 (2.7 to 5.1)	4.4 (2.4 to 6.5)		1.5 (−0.9 to 3.9)	52.3 (−26.6 to 198.5)
White	2.5 (2.5 to 2.6)	2.6 (2.6 to 2.7)	2.8 (2.7 to 2.8)	2.7 (2.6 to 2.8)		0.2 (0.1 to 0.3)	7.2 (4.2 to 10.3)
Hispanic or Latino	2.4 (2.2 to 2.6)	2.4 (2.2 to 2.6)	2.6 (2.4 to 2.9)	2.8 (2.5 to 3.1)		0.4 (0.1 to 0.7)	16.1 (2.3 to 31.3)
**Education**							
Less than HS diploma	4.4 (4.2 to 4.5)	4.7 (4.5 to 4.9)	4.8 (4.6 to 5.0)	5.2 (4.8 to 5.4)	<0.01	0.8 (0.4 to 1.1)	18.2 (9.8 to 27.2)
HS diploma or GED	2.9 (2.8 to 3.0)	3.1 (3.0 to 3.2)	3.3 (3.2 to 3.4)	3.3 (3.2 to 3.4)		0.4 (0.2 to 0.5)	11.9 (6.7 to 17.4)
Some college	2.6 (2.5 to 2.7)	2.7 (2.6 to 2.8)	3.0 (2.8 to 3.1)	2.9 (2.8 to 3.0)		0.4 (0.2 to 0.5)	13.6 (8.3 to 19)
College degree or higher	1.6 (1.6 to 1.7)	1.6 (1.6 to 1.7)	1.7 (1.6 to 1.8)	1.7 (1.6 to 1.8)		0.1 (0 to 0.2)	5.4 (−0.4 to 11.3)

**Abbreviations:** AI/AN = American Indian or Alaska Native; GED = general educational development certificate; HS = high school; NH/PI = Native Hawaiian or Pacific Islander.

* Age-standardized to the 2000 U.S. Census Bureau standard population using age groups 18–44, 45–64, and ≥65 years.

^†^ Respondents were asked, “Has a doctor, nurse, or other health professional ever told you that you had a stroke?” Refused, “don't know,” and missing responses were excluded from analyses.

^§^ Sampling weights were adjusted to reflect 3-year increments (divided by 3).

^¶^ p-values calculated from Wald chi-square tests.

** Persons identified as Hispanic or Latino (Hispanic) might be of any race but are categorized as Hispanic. All racial groups are non-Hispanic.

From 2011–2013 to 2020–2022, stroke prevalence increased 14.6% among adults aged 18–44 years, 15.7% among those aged 45–64 years, 9.3% among women, and 6.2% among men. Among Black, White, and Hispanic adults, stroke prevalence increased by 7.8%, 7.2%, and 16.1%, respectively. The largest percent increase (18.2%) occurred among adults with less than a high school education. DC had a statistically significant decrease in stroke prevalence (19.2%). A statistically significant increase occurred in 10 states (California, Colorado, Minnesota, Mississippi, North Carolina, North Dakota, Ohio, Oklahoma, Tennessee, and West Virginia); the largest increases were in Ohio (20.9%) and Tennessee (20.7%) (Table 2) (Figure). States with a stroke prevalence in the highest quantile during 2020–2022 were predominantly located in southern states. Analyses did not demonstrate significant changes in the prevalence of self-reported stroke during the COVID-19 pandemic compared with before the pandemic (CDC, unpublished data, 2023). 

**TABLE 2:** Age-standardized prevalence* of stroke among noninstitutionalized adults aged ≥18 years,^†^ by jurisdiction — Behavioral Risk Factor Surveillance System, United States, 2011–2022^§^

Jurisdiction	% (95% CI)				Change from 2011–2013 to 2020–2022	
	2011–2013	2014–2016	2017–2019	2020–2022	Percentage point (95% CI)	% (95% CI)
Alabama	4.3 (3.9 to 4.6)	4.2 (3.9 to 4.5)	4.5 (4.2 to 4.9)	4.1 (3.6 to 4.5)	−0.2 (−0.8 to 0.3)	−4.9 (−16.7 to 8.3)
Alaska	2.7 (2.4 to 3.1)	2.4 (2.0 to 2.7)	2.3 (2.0 to 2.7)	2.7 (2.3 to 3.1)	0 (−0.6 to 0.5)	−0.8 (−18.6 to 21.6)
Arizona	2.7 (2.4 to 3.1)	2.8 (2.5 to 3.0)	2.9 (2.6 to 3.2)	2.7 (2.5 to 3.0)	0 (−0.4 to 0.4)	0.6 (−13.4 to 17.8)
Arkansas	3.8 (3.4 to 4.2)	4.1 (3.6 to 4.5)	4.2 (3.8 to 4.6)	3.9 (3.5 to 4.2)	0 (−0.5 to 0.6)	0.9 (−11.7 to 16.2)
California^¶^	2.2 (2.1 to 2.4)	2.4 (2.2 to 2.6)	2.3 (2.1 to 2.5)	2.6 (2.3 to 2.9)	0.4 (0 to 0.7)	17.1 (1.2 to 34.8)
Colorado^¶^	1.9 (1.7 to 2.0)	2.1 (1.9 to 2.2)	2.0 (1.8 to 2.1)	2.2 (2.0 to 2.4)	0.3 (0.1 to 0.6)	16.2 (2.3 to 31.6)
Connecticut	2.0 (1.8 to 2.2)	2.3 (2.1 to 2.5)	2.2 (2.0 to 2.4)	2.2 (1.9 to 2.4)	0.1 (−0.2 to 0.5)	6.4 (−8.8 to 24.1)
Delaware	3.0 (2.7 to 3.4)	2.8 (2.5 to 3.1)	3.1 (2.8 to 3.4)	3.0 (2.6 to 3.4)	0 (−0.5 to 0.5)	−0.4 (−16.5 to 18.3)
District of Columbia^¶^	3.6 (3.1 to 4.0)	3.5 (3.0 to 3.9)	3.6 (3.2 to 4.0)	2.9 (2.5 to 3.3)	−0.7 (−1.3 to −0.1)	−19.2 (−32.1 to −3.8)
Florida	3.0 (2.8 to 3.3)	2.8 (2.6 to 3.0)	3.0 (2.8 to 3.3)	2.9 (2.6 to 3.2)	−0.1 (−0.5 to 0.3)	−3.7 (−16.6 to 10.2)
Georgia	3.2 (2.9 to 3.5)	3.5 (3.1 to 3.8)	3.4 (3.1 to 3.6)	3.4 (3.1 to 3.7)	0.2 (−0.2 to 0.6)	7.7 (−5.3 to 21.6)
Hawaii	2.5 (2.2 to 2.7)	2.6 (2.3 to 2.9)	2.5 (2.3 to 2.8)	2.4 (2.2 to 2.6)	−0.1 (−0.4 to 0.3)	−2.1 (−16.0 to 13.9)
Idaho	2.3 (2.1 to 2.6)	2.4 (2.1 to 2.7)	2.7 (2.3 to 3.1)	2.7 (2.4 to 3.0)	0.3 (−0.1 to 0.7)	14.0 (−2.8 to 35.6)
Illinois	2.7 (2.4 to 3.1)	2.8 (2.5 to 3.1)	2.7 (2.5 to 3.0)	2.9 (2.4 to 3.3)	0.1 (−0.4 to 0.7)	4.4 (−14.9 to 25.7)
Indiana	3.0 (2.8 to 3.2)	3.2 (2.9 to 3.4)	3.4 (3.2 to 3.7)	3.2 (3.0 to 3.5)	0.3 (−0.1 to 0.6)	8.3 (−2.0 to 19.8)
Iowa	2.4 (2.2 to 2.6)	2.3 (2.1 to 2.6)	2.5 (2.3 to 2.7)	2.3 (2.1 to 2.5)	−0.1 (−0.4 to 0.2)	−3.2 (−14.8 to 9.0)
Kansas	2.7 (2.5 to 2.8)	2.7 (2.6 to 2.9)	2.7 (2.6 to 2.9)	2.7 (2.5 to 2.9)	0 (−0.2 to 0.3)	1.4 (−8.1 to 11.9)
Kentucky	3.8 (3.5 to 4.1)	3.9 (3.6 to 4.2)	4.2 (3.8 to 4.6)	4.0 (3.6 to 4.4)	0.2 (−0.3 to 0.7)	5.9 (−6.5 to 19.9)
Louisiana	3.7 (3.4 to 4.1)	3.6 (3.3 to 4.0)	4.4 (4.0 to 4.8)	4.1 (3.7 to 4.4)	0.3 (−0.2 to 0.8)	8.2 (−4.9 to 24.0)
Maine	2.3 (2.1 to 2.5)	2.5 (2.3 to 2.7)	2.9 (2.6 to 3.2)	2.6 (2.4 to 2.8)	0.3 (0 to 0.6)	11.0 (−1.9 to 26.0)
Maryland	2.6 (2.3 to 2.8)	2.7 (2.4 to 2.9)	2.9 (2.7 to 3.1)	2.6 (2.5 to 2.8)	0.1 (−0.2 to 0.4)	3.3 (−7.7 to 15.6)
Massachusetts	2.1 (1.9 to 2.2)	2.4 (2.1 to 2.6)	2.2 (1.9 to 2.5)	2.2 (1.9 to 2.4)	0.1 (−0.2 to 0.4)	4.6 (−9.1 to 19.3)
Michigan	3.1 (2.9 to 3.3)	2.9 (2.7 to 3.1)	3.1 (2.9 to 3.3)	3.0 (2.8 to 3.3)	−0.1 (−0.4 to 0.3)	−1.9 (−12.1 to 9.6)
Minnesota^¶^	2.1 (1.9 to 2.3)	2.1 (1.9 to 2.2)	2.2 (2.0 to 2.3)	2.5 (2.3 to 2.7)	0.4 (0.1 to 0.7)	18.3 (5.2 to 33.9)
Mississippi^¶^	4.0 (3.7 to 4.3)	4.3 (3.9 to 4.7)	4.3 (3.9 to 4.7)	4.7 (4.2 to 5.1)	0.7 (−0.2 to 0.9)	16.8 (4.0 to 30.8)
Missouri	3.1 (2.8 to 3.4)	3.7 (3.4 to 4.0)	3.6 (3.3 to 3.9)	3.3 (3.0 to 3.5)	0.2 (−0.2 to 0.5)	4.9 (−6.9 to 18.1)
Montana	2.8 (2.5 to 3.0)	2.4 (2.2 to 2.7)	2.5 (2.3 to 2.8)	2.5 (2.2 to 2.7)	−0.3 (−0.7 to 0.0)	−11.7 (−23.4 to 1.4)
Nebraska	2.3 (2.2 to 2.5)	2.4 (2.2 to 2.6)	2.5 (2.3 to 2.7)	2.2 (2.0 to 2.4)	−0.1 (−0.4 to 0.1)	−5.6 (−15.8 to 4.7)
Nevada	2.9 (2.5 to 3.3)	2.6 (2.2 to 3.0)	2.9 (2.5 to 3.4)	3.0 (2.5 to 3.6)	0.1 (−0.6 to 0.8)	3.5 (−17.8 to 28.4)
New Hampshire	2.2 (2.0 to 2.4)	2.1 (1.9 to 2.4)	2.2 (1.9 to 2.5)	2.4 (2.1 to 2.7)	0.2 (−0.2 to 0.6)	8.9 (−8.1 to 29.4)
New Jersey	2.2 (2.0 to 2.4)	2.3 (2.1 to 2.6)	2.4 (2.0 to 2.8)	2.4 (2.2 to 2.7)	0.3 (−0.1 to 0.6)	12.1 (−2.7 to 28.3)
New Mexico	2.5 (2.3 to 2.7)	2.7 (2.5 to 3.0)	2.5 (2.2 to 2.7)	2.4 (2.1 to 2.6)	−0.1 (−0.5 to 0.3)	−4.1 (−17.2 to 10.6)
New York	2.2 (2.0 to 2.5)	2.3 (2.1 to 2.5)	2.3 (2.1 to 2.5)	2.2 (2.1 to 2.4)	0 (−0.3 to 0.3)	0.9 (−12.2 to 16.7)
North Carolina^¶^	3.1 (2.9 to 3.3)	3.4 (3.1 to 3.6)	3.5 (3.2 to 3.9)	3.6 (3.2 to 4.0)	0.5 (0 to 0.9)	15.4 (0.1 to 31.3)
North Dakota^¶^	2.2 (1.9 to 2.4)	2.4 (2.1 to 2.6)	2.3 (2.1 to 2.5)	2.6 (2.3 to 2.9)	0.4 (0 to 0.8)	18.5 (0.3 to 40.0)
Ohio^¶^	2.9 (2.7 to 3.1)	3.0 (2.8 to 3.3)	3.3 (3.0 to 3.5)	3.5 (3.3 to 3.8)	0.6 (0.3 to 0.9)	20.9 (9.7 to 33.3)
Oklahoma^¶^	3.2 (3.0 to 3.4)	3.5 (3.2 to 3.8)	4.0 (3.7 to 4.3)	3.8 (3.5 to 4.2)	0.6 (0.2 to 1.0)	18.5 (6.1 to 32.7)
Oregon	2.8 (2.5 to 3.1)	2.7 (2.4 to 2.9)	2.8 (2.6 to 3.1)	2.7 (2.4 to 3.0)	−0.1 (−0.5 to 0.3)	−3.8 (−16.5 to 10.9)
Pennsylvania	2.7 (2.5 to 2.9)	3.0 (2.7 to 3.2)	3.2 (2.9 to 3.5)	3.1 (2.8 to 3.5)	0.4 (0 to 0.9)	15.7 (−0.5 to 32.6)
Rhode Island	2.2 (2.0 to 2.5)	2.2 (1.9 to 2.5)	2.5 (2.3 to 2.8)	2.2 (1.9 to 2.4)	−0.1 (−0.4 to 0.3)	−2.9 (−17.0 to 14.0)
South Carolina	3.3 (3.0 to 3.5)	3.3 (3.1 to 3.6)	3.5 (3.3 to 3.8)	3.3 (2.9 to 3.6)	0 (−0.4 to 0.4)	−0.2 (−11.5 to 12.5)
South Dakota	2.4 (2.1 to 2.7)	2.2 (2.0 to 2.5)	2.3 (2.0 to 2.7)	2.3 (1.9 to 2.7)	−0.1 (−0.6 to 0.4)	−5.6 (−24.8 to 15.6)
Tennessee^¶^	3.5 (3.2 to 3.9)	4.0 (3.7 to 4.3)	4.2 (3.8 to 4.6)	4.2 (3.8 to 4.6)	0.7 (0.2 to 1.3)	20.7 (4.9 to 38.8)
Texas	2.7 (2.4 to 2.9)	2.8 (2.5 to 3.1)	3.4 (3.0 to 3.8)	3.0 (2.7 to 3.3)	0.3 (0 to 0.7)	12.8 (−1.5 to 28.4)
Utah	2.4 (2.2 to 2.5)	2.3 (2.1 to 2.5)	2.4 (2.2 to 2.6)	2.4 (2.2 to 2.6)	0 (−0.2 to 0.3)	0.7 (−9. to 12.4)
Vermont	2.2 (2.0 to 2.4)	2.1 (1.8 to 2.3)	2.2 (2.0 to 2.4)	2.2 (1.9 to 2.4)	0 (−0.4 to 0.3)	−0.7 (−15.3 to 16)
Virginia	2.8 (2.6 to 3.1)	2.7 (2.5 to 2.9)	2.8 (2.6 to 3.1)	2.9 (2.7 to 3.2)	0.1 (−0.2 to 0.4)	3.5 (−8.1 to 16.7)
Washington	2.3 (2.2 to 2.5)	2.6 (2.4 to 2.8)	2.6 (2.4 to 2.8)	2.5 (2.3 to 2.7)	0.2 (−0.1 to 0.4)	6.3 (−4.0 to 17.5)
West Virginia^¶^	3.5 (3.2 to 3.8)	3.9 (3.6 to 4.2)	4.0 (3.7 to 4.4)	3.9 (3.6 to 4.3)	0.5 (0.1 to 0.9)	14.1 (1.5 to 28.3)
Wisconsin	2.2 (1.9 to 2.5)	2.3 (2.0 to 2.6)	2.0 (1.8 to 2.3)	2.5 (2.2 to 2.9)	0.4 (−0.1 to 0.8)	17.3 (−2.7 to 40.9)
Wyoming	2.5 (2.2 to 2.7)	2.7 (2.3 to 3.0)	2.9 (2.5 to 3.2)	2.5 (2.2 to 2.8)	0 (−0.4 to 0.5)	1.4 (−14.6 to 19.7)

* Age-standardized to the 2000 U.S. Census Bureau standard population using age groups 18–44, 45–64, and ≥65 years.

^†^ Respondents were asked, “Has a doctor, nurse, or other health professional ever told you that you had a stroke?” Refused, “don't know,” and missing responses were excluded from analyses.

^§^ Sampling weights were adjusted to reflect 3-year increments (divided by 3).

^¶^ Statistically significant percentage change from 2011–2013 to 2020–2022.

**FIGURE:**
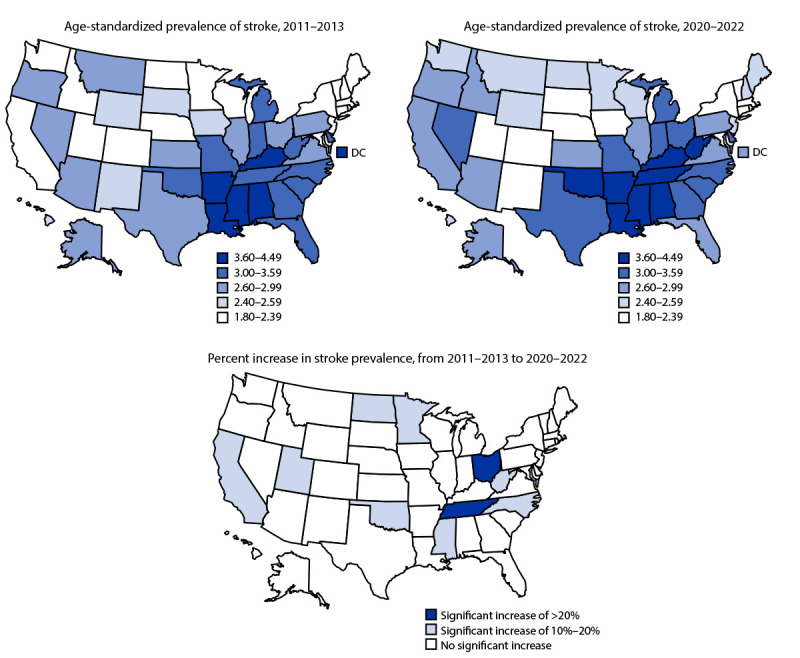
Age-standardized prevalence* of stroke and percentage change among noninstitutionalized adults aged ≥18 years,^†^ by jurisdiction — Behavioral Risk Factor Surveillance System, United States, 2011–2022 **Abbreviation:** DC = District of Columbia. * Age-standardized to the 2000 U.S. Census Bureau standard population using age groups 18–44, 45–64, and ≥65 years. ^†^ Respondents were asked, “Has a doctor, nurse, or other health professional ever told you that you had a stroke?” Refused, “don’t know,” and missing responses were excluded from analyses.

## Discussion

This analysis found that age-standardized stroke prevalence increased by 7.8% from 2011–2013 to 2020–2022. This increase contrasts with the decrease of 3.7% reported during 2006–2010 (*2*). Significant increases in stroke prevalence were observed among several sociodemographic groups, including adults aged 18–44 and 45–64 years; females and males; Black, White, and Hispanic adults; and adults with less than a college degree. Stroke prevalence decreased in DC and had a statistically significant increase in 10 states.

Older age is a known risk factor for stroke (*1*). Stroke prevalence among adults aged ≥65 years was consistent across the study period; however, prevalence among adults aged <65 years increased by approximately 15%. This increase corresponds with a rise of cardiovascular risk factors among younger, working-age adults during recent decades. From 1999–2000 to 2017–2018, obesity prevalence among males increased from 27.5% to 43% and among females from 33.4% to 41.9%; prevalence during 2017–2018 was highest among those aged 40–59 years (44.8%) (*1*). Hypertension prevalence was highest among adults aged 45–64 years, and increased from 40.3% during 1999–2000 to 46.8% during 2017–2018 (*1*). The opioid overdose epidemic^¶^ might also have contributed to increased stroke prevalence among younger adults. A rise in the rate of hospitalizations for stroke among adults aged <45 years during 2006–2015 was associated with opioid use and infective endocarditis and corresponded with the onset of the opioid epidemic (*3*). Among racial and ethnic groups, stroke prevalence was highest among AI/AN, NH/PI, and Black adults. Differences might exist because of a higher prevalence of risk factors among these populations, including comorbid conditions, lower income, and unequal access to health care (*1*,*4*,*5*). In this study, a review of overall and subgroup estimates demonstrated little difference in self-reported stroke prevalence before and during the COVID-19 pandemic (CDC, unpublished data, 2023). However, a previous study demonstrated an increase in stroke mortality rates during the COVID-19 pandemic, with Black adults experiencing a disproportionate increase in excess stroke deaths compared with White adults (*6*). Racial and ethnic disparities, education level inequality, and socioeconomic status disparities within the context of larger structural factors, such as discrimination, might be important to consider when developing focused interventions addressing stroke prevalence (*5*,*7*). Consistent with previous analyses (*2*), states in the highest quantile of stroke prevalence included many in the southeastern United States (a region known as the stroke belt). Increased stroke survival could also contribute to increased stroke prevalence; the rate of thrombolytic therapy for acute ischemic stroke among all racial and ethnic groups increased from 10%–15% during 2003–2009, to 43%–46% in 2021 (*8*). However, disparities persisted, with Asian, Black, and Hispanic patients having lower odds than did White patients of getting to the hospital within 4.5 hours of ischemic stroke onset and of receiving thrombolysis (*8*).

### Limitations

The findings in this report are subject to at least three limitations. First, BRFSS data are self-reported and could be subject to social desirability and recall biases. Second, bias might exist because BRFSS response rates were <50%; however, response rates have been relatively stable during the study period. Finally, as the percentage of acute stroke patients who receive timely thrombolytic treatment increases, an increasing percentage of these patients will likely achieve full recovery.

### Implications for Public Health Practice

Identifying and understanding demographic factors associated with stroke, and disparities in stroke prevalence, might help focus programmatic and clinical interventions to improve the prevention and treatment of stroke at state and national levels. Effective national programs, such as the Million Hearts 2027 initiative,** maintain a repository of sustainable interventions focused on stroke risk factor prevention and improvement in clinical management that can be replicated across diverse communities. For example, the CDC Paul Coverdell National Acute Stroke Program,^††^ a stroke quality-of-care initiative, has demonstrated improvements across the continuum of stroke care among varied geographic and socioeconomic communities. Programmatic and clinical interventions might improve stroke prevention and outcomes, and changes in social determinants of health might also be needed to reduce inequities in stroke prevalence and care (*9*). Such initiatives can include those that promote knowledge of the signs and symptoms of stroke, particularly those which emphasize Act F.A.S.T questions: “Face: Does one side of the face droop when smiling? Arms: Does one arm drift downward when both arms are raised? Speech: Is speech slurred or strange when repeating a simple phrase? Time: If you see any of these signs, call 9-1-1 right away.”^§§^ Acting F.A.S.T is key to stroke survival. Awareness and knowledge of stroke signs and symptoms have increased among US adults, although there is room for improvement (*10*). Better recognition of stroke signs and symptoms might have potentially contributed to increased stroke prevalence, because earlier stroke treatment contributes to improved outcomes (*8*). Advancing focused evidence-based practices and programs for stroke awareness, prevention, and treatment is essential for improving the cerebrovascular health of the nation.

## Footnotes

*
https://www.cdc.gov/nchs/products/databriefs/db456.htm

†
https://www.cdc.gov/brfss/about/index.htm

§ 45 C.F.R. part 46.102(l)(2), 21 C.F.R. part 56; 42 U.S.C. Sect. 241(d); 5 U.S.C. Sect. 552a; 44 U.S.C. Sect. 3501 et seq.

¶
https://aspe.hhs.gov/reports/non-fatal-opioid-overdose-associated-health-outcomes-final-summary-report-0

**
https://millionhearts.hhs.gov/about-million-hearts/index.html

††
https://www.cdc.gov/coverdell/php/about/index.html

§§
https://www.cdc.gov/stroke/signs-symptoms/index.html
